# Could the Induction of Trained Immunity by β-Glucan Serve as a Defense Against COVID-19?

**DOI:** 10.3389/fimmu.2020.01782

**Published:** 2020-07-14

**Authors:** Anne Geller, Jun Yan

**Affiliations:** ^1^Department of Microbiology and Immunology, University of Louisville School of Medicine, Louisville, KY, United States; ^2^Immuno-Oncology Program, Division of Immunotherapy, Department of Surgery, The James Graham Brown Cancer Center, University of Louisville School of Medicine, Louisville, KY, United States

**Keywords:** COVID-19, SARS-CoV-2, trained immunity, β-glucan, innate immunity

## Abstract

As the SARS-CoV-2 virus wreaks havoc on the populations, health care infrastructures and economies of nations around the world, finding ways to protect health care workers and bolster immune responses in the general population while we await an effective vaccine will be the difference between life and death for many people. Recent studies show that innate immune populations may possess a form of memory, termed Trained Immunity (TRIM), where innate immune cells undergo metabolic, mitochondrial, and epigenetic reprogramming following exposure to an initial stimulus that results in a memory phenotype of enhanced immune responses when exposed to a secondary, heterologous, stimulus. Throughout the literature, it has been shown that the induction of TRIM using such inducers as the BCG vaccine and β-glucan can provide protection through altered immune responses against a range of viral infections. Here we hypothesize a potential role for β-glucan in decreasing worldwide morbidity and mortality due to COVID-19, and posit several ideas as to how TRIM may actually shape the observed epidemiological phenomena related to COVID-19. We also evaluate the potential effects of β-glucan in relation to the immune dysregulation and cytokine storm observed in COVID-19. Ultimately, we hypothesize that the use of oral β-glucan in a prophylactic setting could be an effective way to boost immune responses and abrogate symptoms in COVID-19, though clinical trials are necessary to confirm the efficacy of this treatment and to further examine differential effects of β-glucan's from various sources.

## Introduction

Throughout evolution, the majority of cellular life (~97%) has existed without a canonical adaptive immune system capable of generating memory responses (1). In fact, until the appearance of jawed fish 500 million years ago, features of adaptive immunity did not exist (2). Despite this, plants, protists, invertebrates and lower animals certainly had a prescient need to protect themselves from recurrent infections. As such, it is known that in these organisms, the innate immune system evolved ways of programming memory-like features in order to non-specifically prevent infection of common pathogens. This protection in plants is known as Systemic Acquired Resistance (SAR), which is responsible for the observation that following inoculation with attenuated organisms, plants benefit from subsequent protection against a myriad of different infectious agents such as fungal, viral and bacterial pathogens (3). While of course the engagement and activation of adaptive immune responses in humans to protect against sinister infectious agents such as the SARS-CoV-2 virus is important, in seeking ways to quickly protect human life, we stand to learn a great deal from our evolutionary immunological origins in memory-like innate immune responses.

The formal principle of TRIM in humans has been recognized for almost a century, where the first recognized study of TRIM came from Sweden in 1934 and showed that infants given the Bacille Calmette-Guérin (BCG) vaccine against *Mycobacterium tuberculosis* (TB) had an increased survival rate compared to unvaccinated infants, which could not only be attributed to being immune to TB (4). In the late 90s several studies came out that explored the protective effects of β-Glucan, BCG and other vaccines against non-specific secondary pathogens that further supported the concept of TRIM (5–10). More recently, a 2017 study in Denmark showed that early administration of BCG was associated with a reduced mortality rate of 38% within the neonatal period (11). Though the BCG vaccine has gained the most general attention as a known inducer of TRIM, there are several other compounds that also act as potent initiators of TRIM. One such inducer is β-glucan, which is a naturally occurring polysaccharide found in the cell wall of yeast, bacteria and fungi. Like the BCG vaccine, β-glucan is known to induce a phenotype of TRIM, though the mechanism of action is known to be different from BCG.

Following exposure to β-glucan, innate immune cells undergo epigenetic reprogramming that results in cellular activation, augmented cytokine production, and changes in metabolic function that include increased aerobic glycolysis in addition to dose-dependent changes in oxidative phosphorylation (12, 13). Alterations in histone methylation and acetylation are important epigenetic alterations that occur which are responsible for the positive regulation of gene expression. When these “trained” cells then come into contact with heterologous secondary stimuli they are programmed to produce a more robust immune response (14, 15). Accordingly, studies have shown that following treatment with β-glucan, mice were more resistant to bacterial infections such as *Staphylococcus aureus* (16) and parasitic infections such as *Leishmania braziliensis* (17). Importantly, β-glucans of various sources have also been widely shown to have significant anti-viral effects, and have been shown to decrease the severity of both upper and lower respiratory tract viral infections (18–24). We posit that these anti-viral effects could likely be due to the induction of TRIM, though more definitive research is needed to determine whether the general immune stimulatory effects of β-glucans or the induction of TRIM is directly responsible.

As of June 24, 2020, 9.4 million people have been diagnosed with a confirmed case of COVID-19, hundreds of thousands of people have been hospitalized, and over 481,000 people have died worldwide. COVID-19 has presented the modern world with a challenge that global health-care infrastructures have not seen in over a century since the 1918 Spanish influenza pandemic. Though there are several promising vaccine candidates on the horizon, it cannot be expected that a vaccine against SARS-CoV-2 will bring any proximate relief, which indicates that in the interim, it is necessary to focus on effective and easily deployed therapeutics to increase immunity against SARS-CoV-2. Accordingly, several studies have been quickly initiated to investigate whether the induction of TRIM, through the administration of the BCG vaccine, can help protect against COVID-19. On March 30, 2020, the BRACE trial was initiated in Australia, which aimed to give the BCG vaccine to up to 4,170 healthcare workers in order to determine if BCG vaccination can reduce the incidence and severity of COVID-19 during the 2020 pandemic. Due to the excitement and promise of this trial, on May 3, 2020, the Bill and Melinda Gates Foundation gave a 10-million-dollar grant to expand this trial to 10,000 healthcare workers. In support of this study, one epidemiological investigation by Miller et al., has shown a correlation between the universal BCG vaccination policy and reduced morbidity and mortality due to COVID-19 (25).

While the excitement regarding the use of BCG as a prophylactic treatment for COVID-19 is warranted, considering that β-glucan can be administered orally, has an extremely high safety profile, does not require a person to access healthcare to receive the treatment, and is known to act similarly to the BCG vaccine in terms of augmenting innate immune responses, there is a strong argument to be made in favor of the use of β-glucan to prophylactically treat against COVID-19 as well. Here-in, we will highlight the known anti-viral impacts of β-glucan, review the known mechanisms of β-glucan-induced TRIM that could lead to protection against COVID-19, and present our personal view about the immune response to SARS-CoV-2 in the scope of TRIM. Additionally, though there is strong evidence to support the use of β-glucan as an anti-viral agent, COVID-19 has presented with a unique clinical course that involves the development of cytokine storm and thromboembolic events which often lead to mortality. As such, it is also important to consider that the immunostimulatory effects of β-glucan could be detrimental to the subset of patients who do develop cytokine storm and hyperinflammation, and so further research and understanding of the anti-viral mechanisms of β-glucan are needed before conclusions are made, which will also be discussed.

## Natural Compound β-Glucan

### Overview

β-Glucans are a heterogenous group of polysaccharides found abundantly in the cell walls of yeast, bacteria and fungi. They are made of glucose molecules linked together by (1–3), (1–4) or (1–6) β-glycosidic bonds, with varying branching structures coming off of the linear backbone. Despite the rich diversity of glucan structures, only β-glucans that consist of a β(1, 3) linked D-glucose backbone with β(1, 6) branching side chains are classified as biological response modifiers, and are known to have immunogenic properties (26, 27). The majority of these immunogenic β-glucans are purified from fungus and yeast. Importantly, unlike other natural products, β-glucans preserve their bioactivity even after oral digestion (28). In the human diet, β-glucans are abundantly found where oat, barley, wheats, yeasts, and certain mushrooms are rich sources of β-glucan. One cooked cup of oatmeal can have up to 2 mg of β-glucan, however for reference, therapeutic oral doses of β-glucan can contain up to 500 mg (29). Orally administered β-glucan is thought to mediate immunogenicity through receptor-mediated interactions with M cells which translocate luminal immunogens into Peyer's patches, which then interact with resident macrophages and dendritic cells (DCs) (30). Another mechanism is through direct interaction of β-glucan and DCs in Peyer's patches whose projections may extend through the apical epithelial cells and into the intestinal lumen (31, 32). Once β-glucans reach gastrointestinal macrophages, they will travel through the bloodstream or lymph system to target the bone marrow, spleen and lymph nodes (33).

There have been several routes of administration studied regarding β-glucan that include oral, intra-muscular (IM), intra-venous (IV), intra-nasal (IN), and intra-peritoneal (IP) administration. A particular challenge to research on β-glucan is the relative diversity of route of administration, which can lead to very different effects. While in animal studies IM and IP administration are relatively simple, in a human population these routes could be considered too invasive. For this reason, the majority of human studies conducted using β-glucan have used oral β-glucan. As discussed above, oral administration β-glucan is shown to exert immunogenic properties, however it is likely that the systemic administration of β-glucan through either IV or IM routes would result in more pronounced effects. Weighing the immuno-stimulatory function vs. the ease and safety of administration is certainly important, however in this context further studies are needed to determine the best approach (34).

## Known Anti-Viral Properties of β-Glucan

### Antiviral Properties of β-Glucan in Animal Studies

Along with the long list of anti-pathogenic bacterial properties, β-glucan has also shown promising anti-viral properties (19–21, 35). With regards to relevance to COVID-19, β-glucan has shown marked efficacy in abating viruses that impact the upper and lower respiratory tracts and those that culminate in a viral pneumonia. For example, one study showed that the administration oral β-glucan to pigs 3 days prior to infection with swine influenza virus (SIV) decreased the severity of microscopic lung lesions induced by SIV and decreased the detectable SIV nucleic acid present within the lungs days 5, 7, and 10 post-inoculation. Interferon gamma (IFN-γ) and nitric oxide (NO) levels were significantly increased in the bronchoalveolar lavage fluid from the β-glucan treated pigs (20). Enhanced anti-influenza properties have also been observed in mice that have been administered β-glucan, where Vetvicka et al., showed that a 2-week regimen of oral of β-glucan resulted in decreased mortality due to influenza infection. The suppression of phagocytosis is a well-known feature of influenza infections, which significantly contributes to disease pathogenesis, and importantly, this study showed that β-glucan increased the phagocytic capacity of neutrophils (36). β-Glucan was also shown to increase the production of IL-1β, TNF-α, and IFN-γ in peripheral blood, and potentiated the antibody response to influenza infection as compared to controls. Viral titers were shown to be significantly reduced after day 1 post-infection, with viral levels shown to be specifically lowered in heart tissues (19). In agreement with these studies, reports show that in addition to enhanced cytokine functions, a potential mechanism of increased protection from upper and lower respiratory viral infections could be due to increased number, phagocytic capacity and lysosomal enzymatic activity of alveolar macrophages (AMs) (37). These changes to the function and number of AMs may play a very important role in effective viral clearance within the lungs. A study conducted by Medina-Gall et al. that used zebrafish intraperitoneally injected with β-glucan and then subsequently challenged with spring viremia of carp virus (SVCV), a deadly virus that causes significant mortality in carp populations, supported this. Here they showed that β-glucan treated fish exhibited a significant increase in survival at 14 days post-treatment (23, 38, 39).

### Antiviral Properties of β-Glucan in Human Studies

Human studies confirm these findings in animals, where yeast (1, 3)-(1–6) β-glucan was shown to decrease the severity of physical symptoms of upper respiratory tract infections (URTI) (24). This study was also shown to decrease the systolic and diastolic blood pressure of participants receiving β-glucan. This may have specific implications for the use of β-glucan in the setting of COVID-19, as patients with the most severe symptoms requiring intensive care unit (ICU) treatment were shown to have significantly increased blood-pressure compared to those not needing ICU care (40). Another study using β-glucan from the *Pleurotus ostreatus* mushroom significantly reduced the incidence of lower respiratory tract infections and the frequency of the flu and flu-like disease in children (18). A study in older adults age 50-70 who received a β-glucan supplement for 90 days exemplified the protective effects of β-glucan in this high-risk group. Here the number of days that a patient experienced symptoms of a URTI was decreased. The blood from treated individuals also showed increased IFN-γ (35). Finally, in two double-blind, randomized, placebo-controlled studies, orally administered yeast-derived β-glucan was shown to significantly reduce the number of common cold episodes by 25% and led to a milder progression of severe common cold episodes (41, 42). Though of course the symptoms and outcomes of COVID-19 are known to be far more severe than a “common cold” there is evidence here that the administration of β-glucan could lead to a decrease in the severity and an improvement of outcomes, especially in the most vulnerable populations.

It must be noted that in these animal and human studies, β-glucan is shown to impact the immune response which likely benefits anti-viral responses, but it not examined whether these effects are a result of TRIM or a result of β-glucan directly stimulating immune cells which leads to better viral control. Moving forward, studies using β-glucan in viral settings should seek to make this important distinction. This is especially important because if a TRIM-mediated mechanism is at play, the use of β-glucan as a prophylactic would be the indicated clinical course, however if it is due to direct immuno-stimulatory effects, β-glucan could be used as a therapeutic.

## Trained Innate Immunity (TRIM)

### What Is TRIM?

While β-glucan itself causes direct stimulation of immune responses, β-glucan has also been known to act as a training agent which results in amplified immune responses when these trained cells are exposed to a secondary, heterologous, stimulus. Evolutionarily speaking, living multicellular organisms have long been fighting off fungal and bacterial pathogens, and so overtime, it makes sense that organisms lacking adaptive responses would devise a way to protect themselves against these repeated infections. That anti-fungal and bacterial TRIM was likely retained in higher vertebrates, resulting in the TRIM observed following administration of β-glucan or other elements that resemble fungal and bacterial antigens.

Animal studies using β-glucan support the paradigm of TRIM, where exposure to β-glucan followed by a secondary infection with *Staphylococcus aureus* results in protection against the pathogen (5). As Netea et al. points out in his excellent recent review article on TIRIM, models of TRIM using various training agents have shown protection against a host of relevant lethal pathogens such as *Streptococcus pneumonia, Toxoplasma gondii, Escherichia coli*, and rotavirus (43–46). Further, the various examples of the BCG vaccine and β-glucan affording protection against secondary infections, such as *Candida albicans*, in a macrophage specific manner, ultimately leads to the idea that the exposure of innate immune cells, specifically myeloid cells, to specific training stimuli results in a non-specific immune protection (7, 9, 17, 47, 48).

Human studies further support the idea that the induction of non-specific immunity following exposure to an unrelated primary pathogen is driven by innate immune cells. For example, the presence of a latent herpesvirus infection has been shown to protect from future infections against *Listeria monocytogenes* and *Yersinia pestis* in a macrophage dependent manner (46, 49). This data holistically points to the concept that by stimulating the immune response with one pathogen, it is possible to fortify it against infection by another. With this understanding, it is possible to take advantage of such immune responses by using a stimulant, such as β-glucan, that does not actually make an individual sick, but does have the benefit of generating primed immune cells that will respond to a host of lethal infections.

### The Mechanisms of TRIM

Innate immune memory primarily involves macrophages and monocytes, though DCs, and Natural Killer cells (NKs) have also been shown to be involved in TI (14, 50, 51). It has been observed that the effects of TRIM can last for weeks to months, which led to the question of whether cells in the periphery were themselves trained, or whether the administration of a training agent such as β-glucan could impact the bone marrow (BM) which may lead to a more lasting TRIM phenotype. Further, considering that many of the cells known to be involved in TRIM are terminally differentiated, and thus unable to pass their phenotype on to their progeny, it was hypothesized that HSCs may be impacted. Accordingly, it was shown that the administration of intraperitoneal β-glucan treatment results in a biased expansion of Lin-Sca1+cKit+ (LSKs) and Multipotent Myeloid Progenitor 3 (MPP3) HSCs in the BM which are skewed toward the myeloid lineage through GM-CSF and IL-1 (52). In mice treated with β-glucan, the induction of a systemic inflammation using LPS resulted in increased responsiveness and cytokine production from these cells that was seen to last for up to 1 month (53). This education and alteration of HSCs in the BM is responsible for the generation of “central” memory which creates a repertoire of innate cells possessing innate immune memory features, which then migrate to peripheral tissues to generate peripheral memory (46, 54).

### Epigenetic Regulation of TRIM Relating to Antiviral Responses

While the molecular mechanisms of TRIM are still being elucidated, data suggests that epigenetic, metabolic, and mitochondrial alterations each play an integral role. In addition to the described pathways of Dectin-1 activation leading to increased cytokine release, activation of the Dectin-1 receptor by β-glucan also causes important changes to the epigenetic status of immune gene promoters. An example of the epigenetic priming induced by β-glucan is that upon Dectin-1 activation, nuclear factor of activated T-cells (NFAT-1) is dephosphorylated, which results in its translocation through the nuclear membrane. NFAT-1 mediates β-glucan-driven epigenetic training by upregulating immune gene-priming long non-coding RNAs (IP-incRNAs) which culminates in increased levels of trimethylation of histone H3 at lysine 4 (H3K4me3) at promoter sites (14, 55). High levels of H3K4me3 are associated with robust levels of gene expression, and so this epigenetic effect results in more vigorous cytokine production upon re-stimulation of β-glucan-primed immune cells (56). Such epigenetic modifications driven by β-glucan result in inflammatory genes that are ideally positioned to be rapidly activated by secondary infections or stimuli, such as a virus.

The anti-viral effects of epigenetic reprogramming due to the induction of TRIM have already been supported in the context of training the immune response with the BCG vaccine, and so it is likely that β-glucan works in the same way. In a study by Arts et al. it was shown that the BCG vaccine protected from experimental viral infection through the induction of genome-wide epigenetic reprogramming and the upregulation of IL-1β (57). An important note about this experiment is that while the authors used the BCG vaccine to induce TRIM, β-glucan driven TRIM also shows epigenetic regulations that lead to an increased production of IL-1β, indicating that it is likely β-glucan administration would have shown similar effects (58, 59). Additionally, in this experiment, an attenuated strain of the yellow fever virus vaccine was used. Yellow fever is a member of the Flavivirus genus, which are a group of single stranded positive sense RNA viruses. Considering that coronaviruses are also positive sense RNA viruses, there is reason to believe that these findings support the idea that β-glucan could be an effective prophylactic for COVID-19.

### Metabolic Regulation of TRIM Relating to Antiviral Responses

Metabolic changes are also a prominent feature of β-glucan induced TRIM, as vital energy metabolites regulate chromatin-modifying epigenetic enzymes, methylation, histone modification, and the position of the nucleosome by acting as substrates and co-factors. Consequently, the energy state of a cell and the metabolic programs that are initiated as a result of β-glucan stimulation tightly modulate the transcription of immunogenic genes (60). The metabolic switch from oxidative phosphorylation toward aerobic glycolysis is a key feature of TRIM, which has been shown to be mediated through the AKT/mTOR/HIF1α pathway (61). Other notable metabolic features of TRIM are a decrease in itaconate, a product of the decarboxylation of *cis*-aconitate, and increased fumarate and mevalonate accumulation through upregulation of the TCA cycle following stimulation with LPS. β-glucan signaling notably inhibits the LPS mediated upregulation of immune-responsive gene-1 (IRG-1), the enzyme that is responsible for itaconate generation, and stimulates the activity of succinate dehydrogenase, leading to increased fumarate production (62). This is critically important as itaconate is known to induce immune tolerance and anti-inflammatory properties in human monocytes (63, 64).

With regards to the impact of this on anti-viral protection, there is evidence that high levels of itaconate and its derivatives inhibit key Type-I interferon production during viral infection (65, 66). Relating this to SARS-CoV-2 infection, there is current research that suggests that SARS-CoV-2 demonstrates significant sensitivity to Type-I interferon signaling (67). There is also evidence that the ability of SARS-CoV-2 to downregulate type I IFN responses is tightly associated with disease severity, and SARS-CoV-2 has been shown suppress type I IFNs in response to viral infection (68, 69). Indeed, it has been shown that stimulation of DCs with fungal β-glucan stimulates IFN-β production, which in turn activates CD8^+^ T-cells and leads to their increased proliferation, and secretion of IFN-γ and Granzyme-B (70). Thus, for these reasons, using β-glucan to metabolically upregulate Type I IFN responses may lead to better overall viral control.

## Discussion of β-Glucan and Trim in the Scope of COVID-19

### The Viral Pathogenesis of SARS-CoV-2

The SARS-CoV-2 virus is known to bind to the angiotensin-converting enzyme-2 (ACE-2) expressed on various tissues including the heart, kidney, bladder, and especially the lung. In the lungs, SARS-CoV-2 binds to ACE 2 expressed on type II alveolar cells to gain entry to the cells (71, 72). Type II alveolar cells themselves will respond to viral infections through the recognition of pathogen associated molecular patterns (PAMPs), which for a ssRNA virus such as SARS-CoV-2, will likely be genomic viral ssRNA or dsRNA. While SARS-CoV-2 is a positive-sense single stranded virus, dsRNA is an obligate intermediate of positive-stranded RNA viruses, which will accumulate during replication cycles and work as a cytosolic PAMP (73). These PAMPs will be recognized through TLR3 or TLR7 endosomal RNA receptors and the cytosolic RNA sensors RIG-I and MDA5. This signaling causes activation and nuclear translocation of the transcription factors NF-κB and IRF3 which cause type I IFN anti-viral responses that are capable of suppressing early stage viral replication (69, 74). It is thought that the epithelial cells are the main source of anti-viral responses in the first 24–48 h of infection, however in order to mount a sustained immune response, it is necessary that these viral signals are carried over into innate immune cells which can then translate these into adaptive immune responses.

There are several mechanisms that are likely responsible for robust macrophage responses to SARS-CoV-2. First, Type II alveolar cells will secrete a host of inflammatory cytokines in response to viral infection such as IL-1β, IL-6, TNF-α, CXCL10, and CCL2 that will act to recruit other inflammatory cells to help abate the viral infection (74). Alveolar macrophages in the lung have also been shown to express ACE 2, which may indicate that they too are susceptible to infection with SARS-CoV-2 and upon being infected will not only present viral epitopes on MHC I and MHC II for CD8^+^ and CD4^+^ recognition, but will also activate anti-viral IFN type I signaling (75, 76). It is also probable that viral infection of type II pneumocytes results in their eventual apoptosis, which leads to subsequent phagocytosis of these cells by macrophages, resulting in another important mechanism of antigen uptake (77). Further relaying the vitally important role of innate immune cells in responses to SARS-CoV-2, one recent study used single cell RNA sequencing to identify novel receptors of SARS-CoV-2 to understand which immune cells come into contact with SARS-CoV-2 infected cells. This study indicated that macrophages most frequently communicate with the targets of SARS-CoV-2 through chemokines and phagocytic signaling (78). Such studies indicate that the ability of innate immune cells to survive infection with SARS-CoV-2 and maintain the capacity to educate adaptive responses is vital for successful protection.

### Innate Immune Responses in COVID-19

Information about the nature of the SARS-CoV-2 virus and the related immune responses are still emerging, and many aspects of the viral pathogenesis are still unknown. Interestingly, there seems to be a dynamic role for immune responses, where a lack of competent Th1 adaptive immune responses and decreased CD4^+^ and CD8^+^ T-cells, resulting in lymphopenia, have been observed in some patients with the most severe disease, while at the same time, overly robust immune responses leading to cytokine storm are also being observed in the most severe cases (79–81). An interesting hypothesis to explain this could be that innate immune responses are critical in early stages, however their most important role is actually in their ability to swiftly and energetically activate Th1 type adaptive responses. When macrophages and DCs fail to galvanize and educate T-cell and B-cell activation, they continue to aberrantly secrete cytokines such as IL-6 and TNFα in efforts to control viral infection, however this results in cascading inflammation, eventually resulting in cytokine storm. This hypothesis would be consistent with observed clinical data of increased IL-6 and TNFα in patients with the most severe responses (82, 83). Our hypothesis is strongly supported by work from Zhao et al., who showed that in mice infected with SARS-CoV, severe disease was correlated with slow kinetics of viral clearance and delayed activation and transit of respiratory DCs to the draining lymph nodes, leading to deficient virus-specific T-cell responses. They also showed that an inhibitory subset of alveolar macrophages prevented the development of immune responses, which could be reversed by giving a treatment, poly I:C, that stimulates TLR3 activation and leads to cellular activation of AMs and DCs (84). While this research relates to SARS-CoV and not SARS-CoV-2, the viruses are known to share a relatively high degree of sequence homology, so there is reason to believe that similar mechanisms are at play between the two viruses due to their similar viral structure (85).

A recent publication by Zhang et al. utilized bronchoalveolar lavage fluid (BALF) from healthy controls and patients with both mild and severe COVID-19 to perform single-cell RNA sequencing. In their model, they identified four groups of human macrophage subsets in the lung and tracked how these changed in COVID-19. Interestingly, they found that AMs, defined by transcriptomics and expression of FABP4, were significantly decreased in COVID-19 infection as compared to healthy controls, and more significantly depleted in severe infections as compared to mild ones. This indicates that the function and presence of AMs are specifically impacted due to SARS-CoV-2, and that their presence likely plays a critical role in protecting against the progression of symptoms (86). Yao et al. have shown that AMs can be targeted for training, and other studies have shown that following β-glucan treatment, AMs in the lung show enhanced IL-1 production and phagocytic properties (37, 87). Though the ability of β-glucan to specifically induce TRIM in alveolar macrophages has not been shown, β-glucan has been shown to enhance cellular activity, cytokine production and phagocytosis in alveolar macrophages, indicating that TRIM may be involved (88).

Taking this into consideration, we pose that in addition to the general immunological benefits of β-glucan, the mechanism of SARS-CoV-2 and related immune responses highlights a very relevant and specific role for β-glucan, as it is known to impact innate immune cells in such a way that they not only are more effective at fighting initial infections, but that they are also better at activating adaptive immune responses. As a result of TRIM induced by β-glucan, we hypothesize that macrophages and DCs would have increased phagocytic capacity, which could not only lead to better viral control, but also to better processing and presentation of viral particles on MHCs (26). Trained macrophages could also elicit enhanced NK cell and neutrophil function. It is also known that β-glucan polarizes tolerogenic M2 macrophages to an M1 phenotype, which would result in increased activation and cytokine secretion, and increased propagation of Th1 T-cell responses (89). Adding to this enhanced activation, it has also been shown that autocrine type I IFN signaling in DCs stimulated with fungal β-glucan promotes antigen presentation to CD8^+^ T-cells, which in the context of the paper written by Zhao et al., could be an extremely important way to boost immune responses against SARS-CoV-2 (70). There is also evidence that β-glucan treated and trained DCs are more efficient at supporting B-cell responses and the production of neutralizing antibodies, which further helps to transition the early innate immune response toward a long-lasting, hyper specific adaptive response (90). We ultimately theorize that the activation of macrophages, DCs, NK cell and neutrophils due to TRIM induced by β-glucan may result in more effective initial responses to infection, enhanced T and B-cell responses against SARS-CoV-2, and an overall decrease in the duration and severity of symptoms in COVID-19.

As previously mentioned, while the induction of robust innate immune responses should generally benefit anti-viral processes, COVID-19 has posed a specific challenge to clinicians due to the development of a hyperinflammatory state marked by increased serum levels of inflammatory chemokines and cytokines, that is a major cause of disease severity and death (40, 79, 91, 92). Like other corona viruses, SARS-CoV-2 has been shown to result in respiratory failure due to local hyperinflammation and ARDS, which has been linked to Macrophage Activation Syndrome (MAS) (93–95). Patients with severe disease have been shown to have increased levels of IL-6, TNFα, MCP1, MIP1A, and IP10, which is also correlated with endothelial dysfunction and increased levels of D-dimer (96). Contrastingly, patients with moderate disease that experience mild symptoms and quickly recover from infection are known to show only modest increases in serum cytokines (97). Taking all of this information together, it is likely that as postulated above, rapid and efficacious initial immune responses are essential for control of viremia, however when these mechanisms fail, dysregulated immune responses prevail resulting in hyper-inflammation and rapid decompensation. For this reason, using an immunostimulant such as β-glucan in later stages of disease could be inappropriate, and could further exacerbate disease. In this setting, therapeutics that quell the immune response such as inhibitors of IL-6 and TNFα would be most appropriate and have shown some degree of clinical promise (98, 99).

Taking this together, we postulate that β-glucan would be best used in the prophylactic setting, where it could utilize processes of TRIM to prime innate immune cells and help to fortify the initial immune responses in the general population to prevent potential SRAS-CoV-2 infection. It could also contribute to a decrease in symptoms in mild and moderate patients. It cannot be ruled out however, that pre-treatment with β-glucan could further exacerbate the already severe hyperinflammation that develops in some patients. Therefore, clinical trials are needed to determine the safety profile and the efficacy of β-glucan in the prophylactic anti-viral setting.

### Exploring the Age Demographics of COVID-19 in Relation to TRIM

Another interesting facet of COVID-19 is that age bears a strong negative association with disease severity, where children, especially those under 18, do contract COVID-19 but see relatively few immediate serious adverse effects (100, 101). Though children rarely develop ARDS due to COVID-19, recent reports suggest that COVID-19 is related to the development of a Kawasaki disease-like syndrome in the pediatric population. There are several theories that have been posed to explain why older adults have the highest mortality rate. There are two potential theories that we could like to explore here. The first, is that as stated above, the ability of innate immune cells to educate adaptive immune responses is the critical synapse in mounting viral protection against SARS-CoV-2, and when this fails, innate immune responses prevail, resulting in hyperinflammation, and cytokine storm. Around age 20, the thymus begins to erode, resulting in a decreased production of naïve T-cells, and an increased relative ratio of more differentiated T-cell subsets. CD8^+^ T-cells specifically are seen to decline drastically with age due to this thymic loss (102, 103). Incidentally, the rate of CD8^+^ T-cell decline is also more pronounced in men, which could possibly be why men seem to experience worse outcomes due to COVID-19 (104, 105). It can thus be hypothesized that the ability of the innate immune system to educate adaptive immune responses, and the following generation of CD8^+^ T-cells specific for SARS-CoV-2 and the production of neutralizing antibodies by B-cells is significantly reduced in adults, and potentially specifically male adults. While the use of β-glucan would not replenish naïve CD8^+^ T-cells, as discussed above it can aid in the ability of innate cells to uptake antigen and reinforce the potency of presentation to T-cells, which could help improve outcomes for the most at risk.

A second hypothesis as to why children are relatively unscathed during this pandemic relates to the induction of TRIM due to routine vaccination schedules in children, which usually last until age 18. While the BCG vaccination is best associated with the induction of TRIM, there is evidence that childhood immunizations can lead to heterologous non-specific immunological effects, which is likely due to the induction of TRIM (106). As children in the United States do not receive the BCG vaccination, other required vaccines would have to be responsible for these effects. Fittingly, cohort studies of the measles, diphtheria-tetanus, and diphtheria-tetanus-pertussis vaccination are correlated with increased non-specific immunogenicity (107). This, of course, relates to the earlier mentioned findings that in countries where individuals routinely receive the BCG vaccine, there are observed lower mortality rates due to COVID-19. This data is certainly preliminary, however supports the idea that the induction of systemic TRIM can help protect against COVID-19 (108). It will be important to closely monitor the results of the aforementioned clinical trials to see if this correlation holds and can be supported more than just circumstantially. Even more, while the BCG vaccine is extremely useful in preventing TB and even in treating bladder cancer, there can be serious adverse effects which include, but are not limited to, the formation of an injection site abscess, lymphadenitis, severe local reactions, and even death (109–111). Though death due to BCG vaccination is rare, it is shown to be associated with an immunocompromised status (111). As immunocompromised patients are a high-risk group in regard to COVID-19, this indicates that the BCG vaccine could not be used to protect these patients who desperately need to be protected. For these reasons, there lies a strong argument that use of natural compound β-glucan to induce TRIM and to reinforce innate immune responses in a prophylactic setting could be an effective therapeutic, that would carry a relatively lower cost and increased safety profile compared to other interventions such as the BCG vaccination, especially in the at-risk populations.

## Conclusions

Understanding the exact mechanism of the immune response to SARS-CoV-2 will surely guide therapeutic and preventative interventions moving forward. It will also be critically important to understand why some patients develop a hyperinflammatory syndrome as this will shape prevention and treatment strategies. As we work to understand these mechanisms, incipient data is showing that innate immune responses in COVID-19 are essential in mounting a successful immune response and when this process fails, hyperinflammation occurs. β-Glucan has been shown to possess a range of anti-viral properties, and we submit that its role as an inducer of TRIM could possibly aid immune responses against SARS-CoV-2 and could help to prevent severe clinical courses. While we await the development of an effective vaccine, we will need to focus on preventative and therapeutic options that can be safety and quickly implemented to bolster immune responses.

We hypothesize that the use of oral β-glucan in the prophylactic setting may be an efficient, low-cost and safe way to help support this immune response, however clinical research and trials are needed to confirm the safety and efficacy of this treatment, and determine which sources and specific doses of β-glucan may be most effective in this context. Further while oral β-glucan would be the safest route of administration and does show important physiological effects, the method of β-glucan administration must also be further studied. In this regard, we pose that research on this topic is important, and the development of clinical trials to answer these questions are necessary in order to evaluate this potentially important treatment. Additionally, given the development of hyperinflammatory responses in severe COVID-19 patients, exclusion criterion should be considered and implemented. Finally, as we seek to understand the anti-viral mechanisms of β-glucan, it is important to make the distinction between general immunostimulatory effects and effects due to the induction of TRIM. Understanding whether TRIM processes are responsible for anti-viral responses will surely give further insight into other potential anti-viral strategies, as the novel SARS-CoV-2 is not the first, nor will it be the last time the human population must deal with a viral pandemic.

## Data Availability Statement

The original contributions presented in the study are included in the article, further inquiries can be directed to the corresponding author/s.

## Author Contributions

AG contributed to the conceptualization, planning, and writing of the manuscript. JY contributed to the conceptualization, planning, direction, and editing of the manuscript. All authors contributed to the article and approved the submitted version.

## Conflict of Interest

The authors declare that the research was conducted in the absence of any commercial or financial relationships that could be construed as a potential conflict of interest.
